# Supplementary material for: ATG14 targets lipid droplets and acts as an autophagic receptor for syntaxin18-regulated lipid droplet turnover
Source: Nat Commun. 2024 Jan 20;15:631. doi: 10.1038/s41467-024-44978-w (PMC10799895; doi:10.1038/s41467-024-44978-w)
Supplement: Supplementary file 1 — Supplementary Information [file 41467_2024_44978_MOESM1_ESM.pdf]

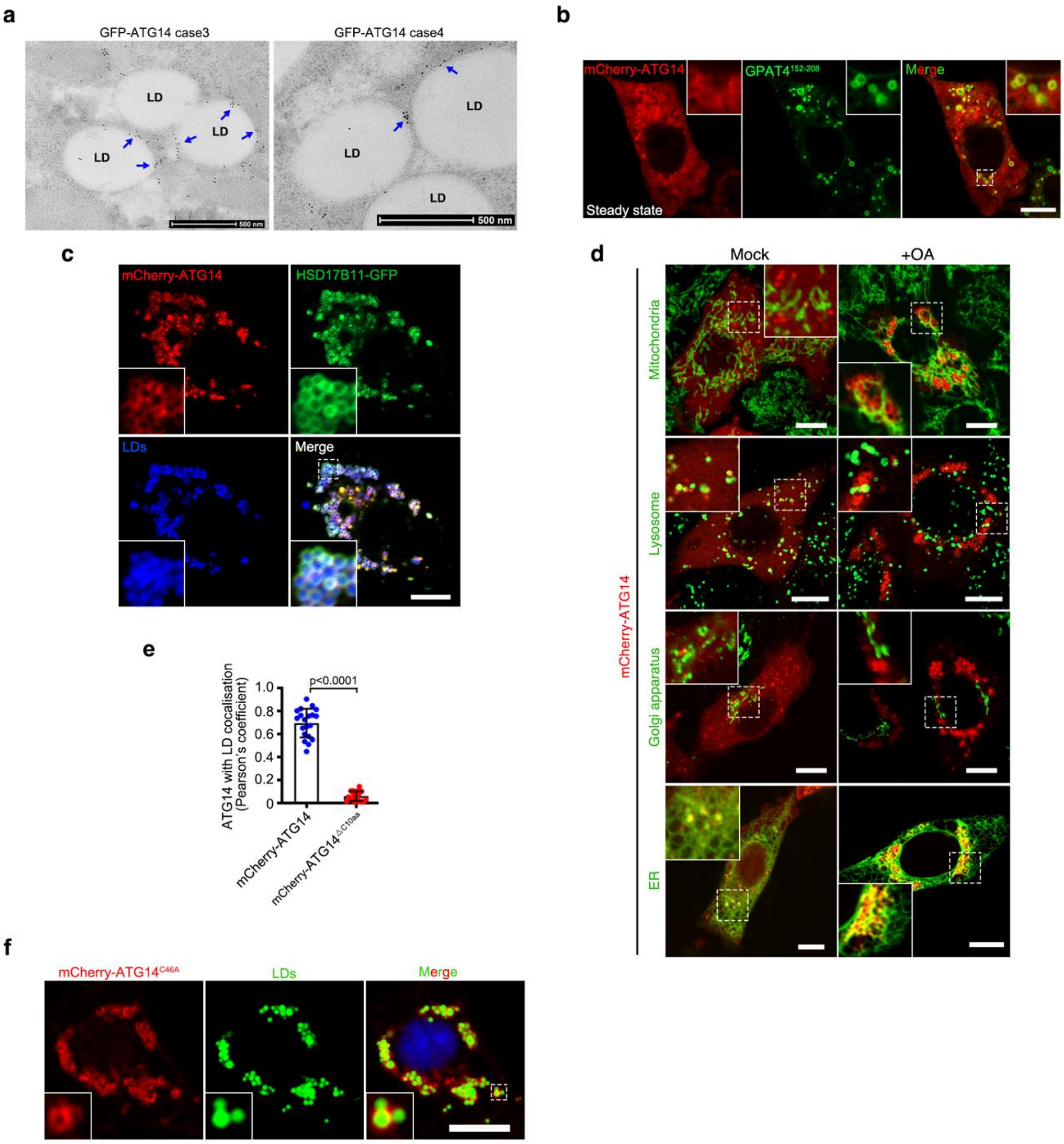
a
GFP-ATG14 case3
GFP-ATG14 case4
LD
500 nm
b
mCherry-ATG14
GPAT4152-208
Merge
Steady state
c
mCherry-ATG14
HSD17B11-GFP
LDs
Merge
d
Mock
+OA
Mitochondria
Lysosome
Golgi apparatus
ER
mCherry-ATG14
e
ATG14 with LD cocalisation
(Pearson's coefficient)
p<0.0001
1.0
0.8
0.6
0.4
0.2
0
mCherry-ATG14
mCherry-ATG14ΔC10aa
f
mCherry-ATG14C46A
LDs
Merge

Figure S1. ATG14 targets LDs.

(a) Representative immune-gold TEM images of cells expressed with GFP-ATG14 and treated with OA for 12 h. Blue arrows mark GFP-ATG14 dots are enriched on the surface of LDs. Scar bar represents 500 nm. **(b)** HeLa cells expressing mCherry-ATG14 and GFP-GPAT4$^{152\text{-}208}$ were fixed. Cells were imaged by confocal microscopy. Scar bar represents 10 μm. **(c)** HeLa cells expressing HSD17B11-GFP and mCherry-ATG14 were treated with 200 μM OA for 12 h, then fixed and labeled the LDs with LipidTOX Deep Red (blue). Cells were imaged by confocal microscopy. Scar bar represents 10 μm. **(d)** Co-localizations between mCherry-ATG14 and mitochondria (anti-Tom20), or Lysosomes (anti-LAMP2) or Golgi (anti-GM130) or ER (GFP-RAMP4) were analyzed via confocal microscopy. Scar bar represents 10 μm. (**e**) Colocalization of mCherry-ATG14 or mCherry-ATG14$^{\triangle 10aa}$ and LDs (Pearson's Coefficient), n=20 cells. Error bars, mean ± SD of three independent experiments. Two-tailed Unpaired Student's t-test. **(f)** HeLa cells expressing mCherry-ATG14^{C46A} were treated with 200 μM OA for 12 h, then fixed and labeled the LDs with BODIPY-493/503 (green). The nuclei were stained with DAPI. Cells were imaged by confocal microscopy. Scar bar represents 10 μm. Source data are provided as a Source Data file.

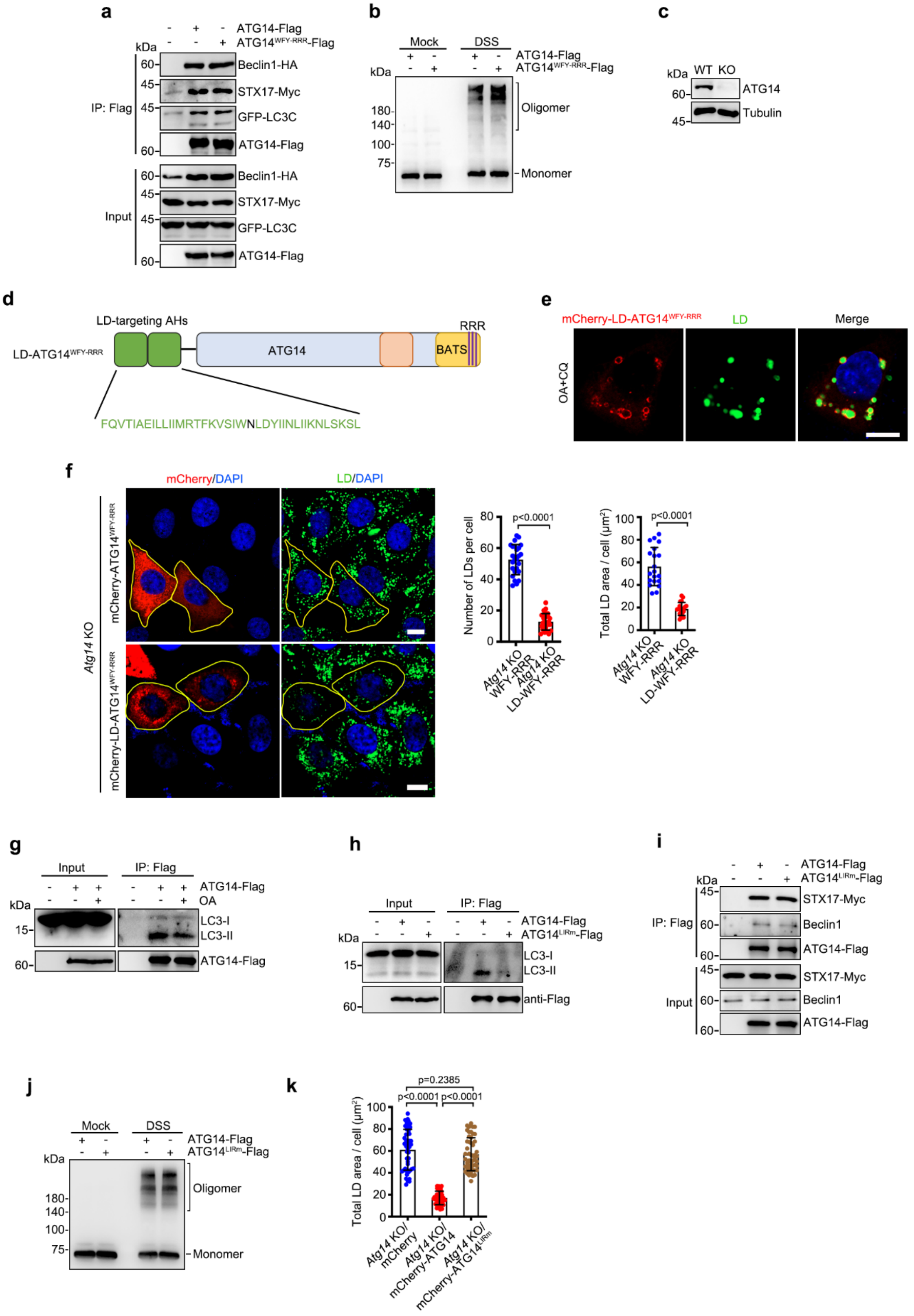
a
ATG14-Flag
ATG14WFY-RRR-Flag
kDa
IP: Flag
Input
Beclin1-HA
STX17-Myc
GFP-LC3C
ATG14-Flag
b
Mock
DSS
Oligomer
Monomer
c
WT KO
ATG14
Tubulin
d
LD-targeting AHs
LD-ATG14WFY-RRR
ATG14
BATS
RRR
FQVTIAEILLIIMRTFKVSIWNLDYIINLIIKNLSKSL
e
mCherry-LD-ATG14WFY-RRR
LD
Merge
OA+CQ
f
mCherry/DAPI
LD/DAPI
Atg14 KO
mCherry-ATG14WFY-RRR
mCherry-LD-ATG14WFY-RRR
Number of LDs per cell
Total LD area / cell (μm2)
p<0.0001
Atg14 KO WFY-RRR
Atg14 KO LD-WFY-RRR
g
Input
IP: Flag
ATG14-Flag
OA
LC3-I
LC3-II
h
ATG14LIRm-Flag
anti-Flag
i
Beclin1
j
k
p=0.2385
Atg14 KO/ mCherry
Atg14 KO/ mCherry-ATG14
Atg14 KO/ mCherry-ATG14LIRm

Figure S2. ATG14 overexpression induces lipophagy.

(a) The Flag tagged ATG14 or ATG14$^{WFY-RRR}$ was co-expressed in HEK293T cells with GFP-LC3C, STX17-Myc and Beclin1-HA. Protein interactions were detected by immunoprecipitation with anti-Flag beads and immunoblotting analysis. **(b)** HEK293T cells were transfected with ATG14-Flag or ATG14$^{WFY-RRR}$-Flag for 36 h and treated with 0.2 mM DSS for 30 min before collecting. Cell lysates were analyzed via western blot. **(c)** *Atg14* wild type and knockout HeLa cells were analyzed via immunoblotting analysis. **(d)** The design of LD-resident ATG14$^{WFY-RRR}$ protein was shown. **(e)** HeLa cells expressing mCherry-LD-ATG14$^{WFY-RRR}$ were treated with OA for 12 h and CQ for 6 h, then fixed and labeled the LDs with BODIPY-493/503 (green). The nuclei were stained with DAPI. **(f)** *Atg14* knockout HeLa cells expressing mCherry-ATG14$^{WFY-RRR}$ or mCherry-LD-ATG14$^{WFY-RRR}$ were treated with 200 μM OA for 6 h, then fixed and labeled the LDs with BODIPY-493/503 (green). The nuclei were stained with DAPI. Cells were imaged by confocal microscopy. Scar bar represents 10 μm. Number (n=25) and total area (n=20) of LDs in each cell was counted from three independent experiments. Error bars, mean ± SD. Two-tailed Unpaired Student's t-test. **(g)** The Flag tagged ATG14 was expressed in HEK293T cells and cells were further treated with or without 200 μM OA for 12 h. Protein interactions between ATG14-Flag and endogenous LC3 were detected by immunoprecipitation with anti-Flag beads and immunoblotting analysis. **(h)** The Flag tagged ATG14 or ATG14^{LIRm} was expressed in HEK293T cells. Protein interactions between ATG14-Flag and endogenous LC3 were detected by immunoprecipitation with anti-Flag beads and immunoblotting analysis. **(i)** The Flag tagged ATG14 or ATG14^{LIRm} was co-expressed in HEK293T cells with STX17-Myc. Protein interactions between ATG14-Flag and STX17-

Myc or endogenous Beclin1 were detected by immunoprecipitation with anti-Flag beads and immunoblotting analysis. **(j)** HEK293T cells were transfected with ATG14-Flag or ATG14^{LIRm}-Flag for 36 h and treated with 0.2 mM DSS for 30 min before collecting. Cell lysates were analyzed via western blot. **(k)** Total area of LDs in each cell in (**Fig. 2n**) was counted from 50 cells of three independent experiments. Error bars, mean ± SD. Two-tailed Unpaired Student's t-test. Source data are provided as a Source Data file.

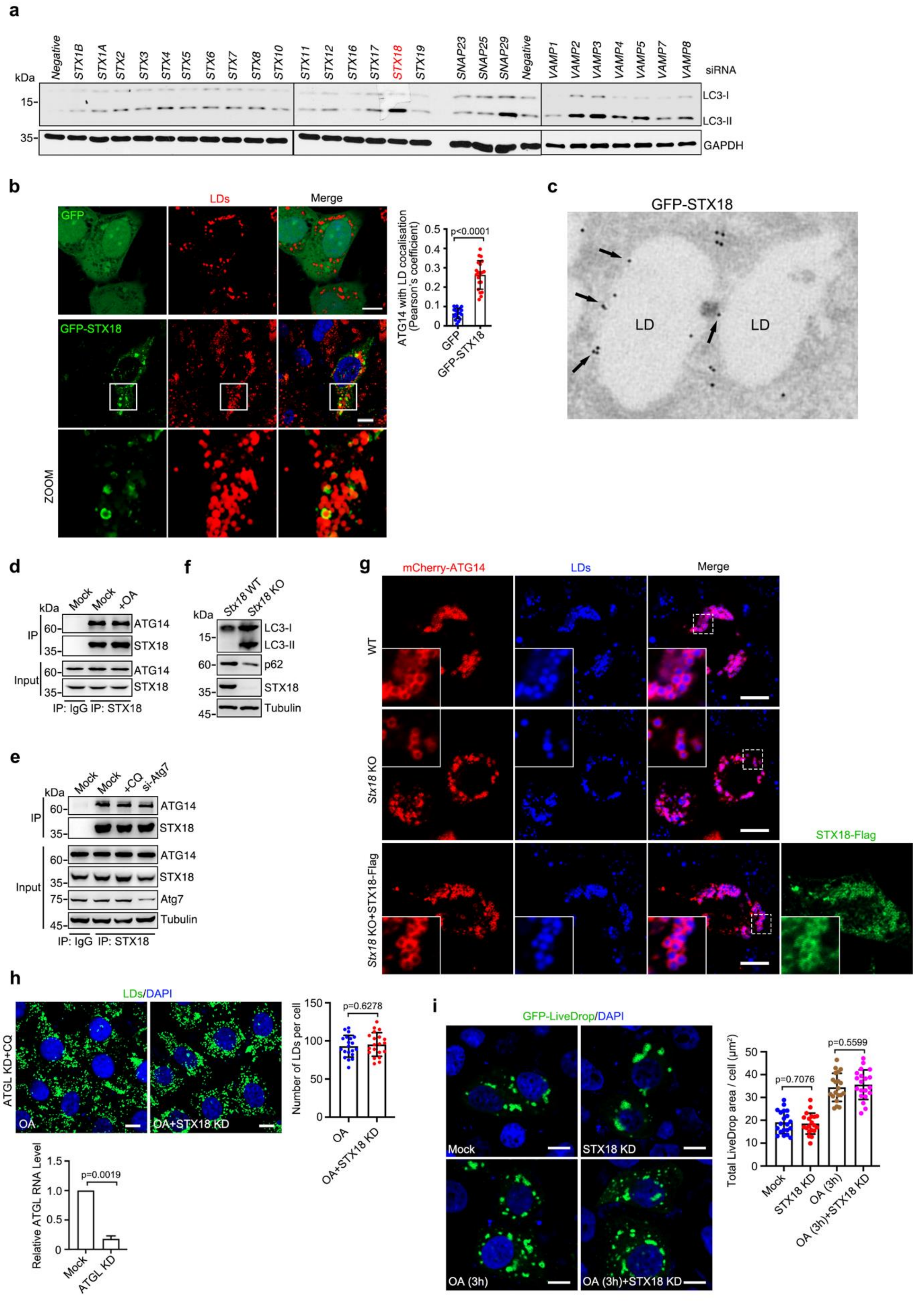
a
b
c
d
e
f
g
h
i
siRNA
Negative
STX1B
STX1A
STX2
STX3
STX4
STX5
STX6
STX7
STX8
STX10
STX11
STX12
STX16
STX17
STX18
STX19
SNAP23
SNAP25
SNAP29
VAMP1
VAMP2
VAMP3
VAMP4
VAMP5
VAMP7
VAMP8
kDa
LC3-I
LC3-II
GAPDH
GFP
LDs
Merge
GFP-STX18
ZOOM
ATG14 with LD cocalisation (Pearson's coefficient)
p<0.0001
LD
Mock
+OA
IP
Input
ATG14
STX18
IP: IgG
IP: STX18
+CQ
si-Atg7
Atg7
Tubulin
Stx18 WT
Stx18 KO
p62
mCherry-ATG14
WT
Stx18 KO+STX18-Flag
STX18-Flag
LDs/DAPI
ATGL KD+CQ
OA
OA+STX18 KD
Number of LDs per cell
p=0.6278
Relative ATGL RNA Level
p=0.0019
ATGL KD
GFP-LiveDrop/DAPI
STX18 KD
OA (3h)
OA (3h)+STX18 KD
Total LiveDrop area / cell (μm²)
p=0.7076
p=0.5599

Figure S3. STX18 targets LDs.

(a) Cells were treated with negative or indicated siRNAs for 48 h. Cell lysates were analyzed via western blot. **(b)** HeLa cells expressing GFP or GFP-STX18 were treated with 200 μM OA for 12 h, then fixed and labeled the LDs with LipidTOX Red (red). The nuclei were stained with DAPI. Cells were imaged by confocal microscopy. Scar bar represents 10 μm. Colocalization of LDs with GFP (20 cells) or GFP-STX18 (20 cells) (Pearson's Coefficient) were analyzed. Error bars, mean ± SD of three independent experiments. Two-tailed Unpaired Student's t-test. **(c)** Low magnification immunogold electron micrograph of GFP-STX18 transfected HepG2 cells. Black arrows mark GFP-STX18 dots are enriched on the surface of LDs. **(d)** HEK293T cells were treated with or without OA for 12 h. Protein interactions were detected by immunoprecipitation with anti-IgG or STX18 antibodies and immunoblotting analysis. **(e)** HEK293T cells were transfected with or without si-Atg7, and treated with or without CQ for 6 h. Protein interactions were detected by immunoprecipitation with anti-IgG or STX18 antibodies and immunoblotting analysis. **(f)** *Stx18* wild type and knockout HeLa cells were analyzed via immunoblotting analysis. **(g)** *Stx18* wild type and knockout HeLa cells co-expressing vector or STX18-Flag with mCherry-ATG14 were treated with 200 μM OA for 12 h, then fixed and labeled the LDs with LipidTOX Deep Red (blue). Cells were imaged by confocal microscopy. Scar bar represents 10 μm. **(h)** HeLa cells were transfected with ATGL or/and STX18 siRNA for 48 h, and treated with 200 μM OA and CQ for 6 h. LDs were labeled with BODIPY-493/503 (green). The nuclei were stained with DAPI. Cells were imaged by confocal microscopy. Scar bar represents 10 μm. Number of LDs in each cell was counted from 20 cells of three independent experiments. qPCR assays confirmed

the efficiency of siRNAs-mediated ATGL suppression in HeLa cells (n=2). Error bars, mean ± SD. Two-tailed Unpaired Student's t-test. **(i)** HeLa cells expressing GFP-LiveDrop were transfected with or without STX18 siRNA for 48 h, and treated with or without 200 μM OA for 3 h. The nuclei were stained with DAPI. Cells were imaged by confocal microscopy. Scar bar represents 10 μm. Total area (n=20) of LiveDrop in each cell was counted from three independent experiments. Error bars, mean ± SD. Two-tailed Unpaired Student's t-test. Source data are provided as a Source Data file.

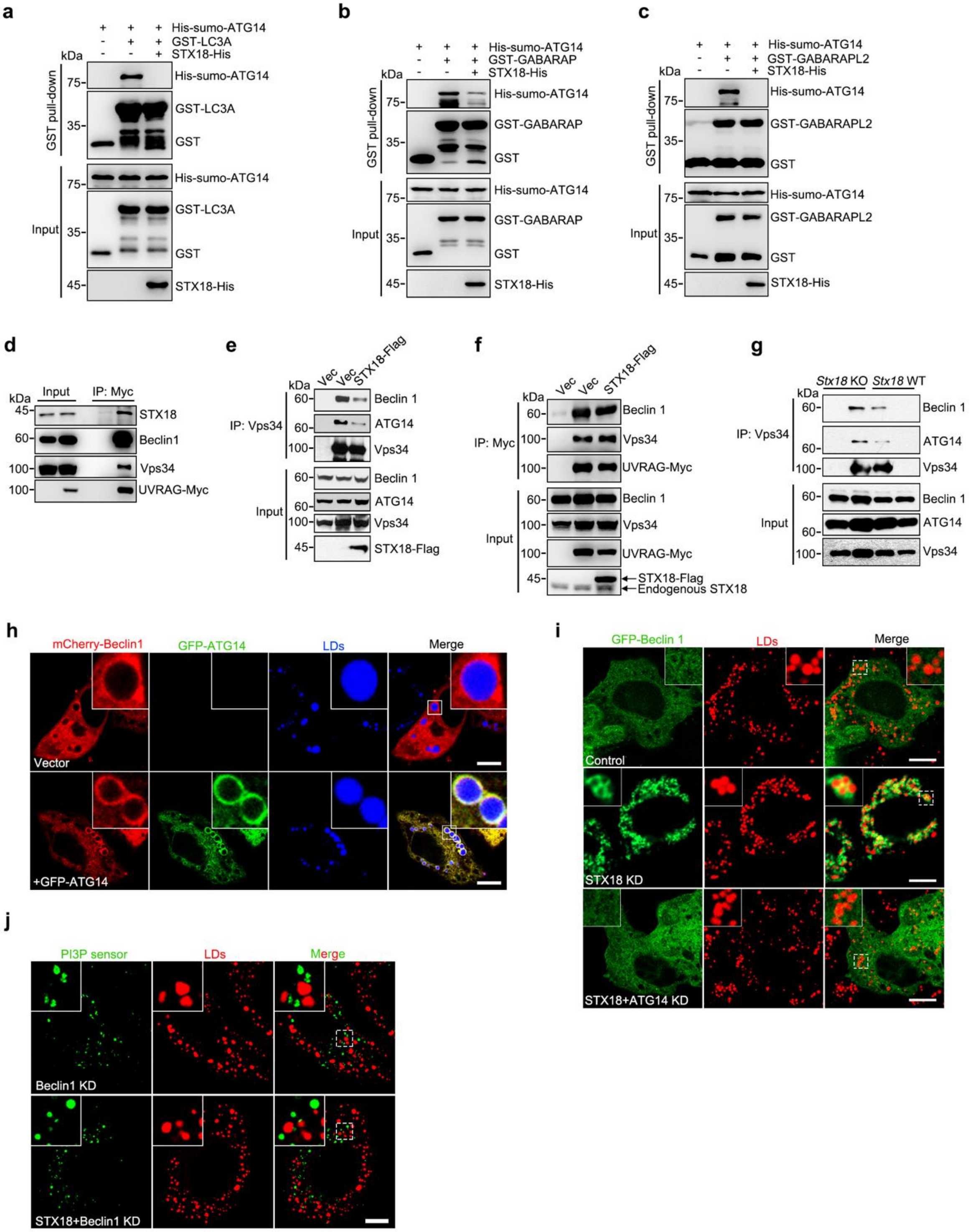
a
His-sumo-ATG14
GST-LC3A
STX18-His
kDa
GST pull-down
Input
GST
b
GST-GABARAP
c
GST-GABARAPL2
d
Input
IP: Myc
STX18
Beclin1
Vps34
UVRAG-Myc
e
Vec
STX18-Flag
Beclin 1
ATG14
IP: Vps34
f
→ Endogenous STX18
g
Stx18 KO
Stx18 WT
h
mCherry-Beclin1
GFP-ATG14
LDs
Merge
Vector
+GFP-ATG14
i
GFP-Beclin 1
Control
STX18 KD
STX18+ATG14 KD
j
PI3P sensor
Beclin1 KD
STX18+Beclin1 KD

Figure S4. STX18 interacts with ATG14 and subverts ATG14-LC3 interaction and acts as a negative regulator of PI3KC3-C1 complex.

(a) The effect of STX18 on the interaction of LC3A with ATG14 was detected by *in vitro* GST pull-down. **(b)** The effect of STX18 on the interaction of GABARAP with ATG14 was detected by *in vitro* GST pull-down. **(c)** The effect of STX18 on the interaction of GABARAPL2 with ATG14 was detected by *in vitro* GST pull-down. **(d)** HEK293T cells were transfected with UVRAG-Myc for 36 h. Protein interactions were detected by immunoprecipitation with anti-Myc beads and immunoblotting analysis. **(e)** HEK293T cells were transfected with or without STX18-Flag for 36 h. Cells were subjected to Vps34 IP and analyzed via western blot. **(f)** HEK293T cells were transfected with UVRAG-Myc with or without STX18-Flag for 36 h. Protein interactions were detected by immunoprecipitation with anti-Myc beads and immunoblotting analysis. **(g)** *Stx18* wild type and knockout HEK293T cells were subjected to Vps34 IP and analyzed via western blot. **(h)** HeLa cells expressing mCherry-Beclin1 with or without GFP-ATG14 were treated with 200 μM OA for 12 h, then fixed and labeled the LDs with LipidTOX Deep Red (blue). Cells were imaged by confocal microscopy. Scar bar represents 10 μm. **(i)** HeLa cells expressing GFP-Beclin1 were transfected with STX18 or/and ATG14 siRNA for 48 h and treated with 200 μM OA for 12 h, then fixed and labeled the LDs with LipidTOX Red (red). Cells were imaged by confocal microscopy. Scar bar represents 10 μm. **(j)** HeLa cells expressing GFP-FYVE$_{SARA}$ were transfected with pSUPER-shBeclin1 or/and STX18 siRNA for 48 h, and treated with 200 μM OA for 12 h. LDs were labeled with LipidTOX Red (red). Cells were imaged by confocal microscopy. Scar bar represents 10 μm. Source data are provided as a Source Data file.

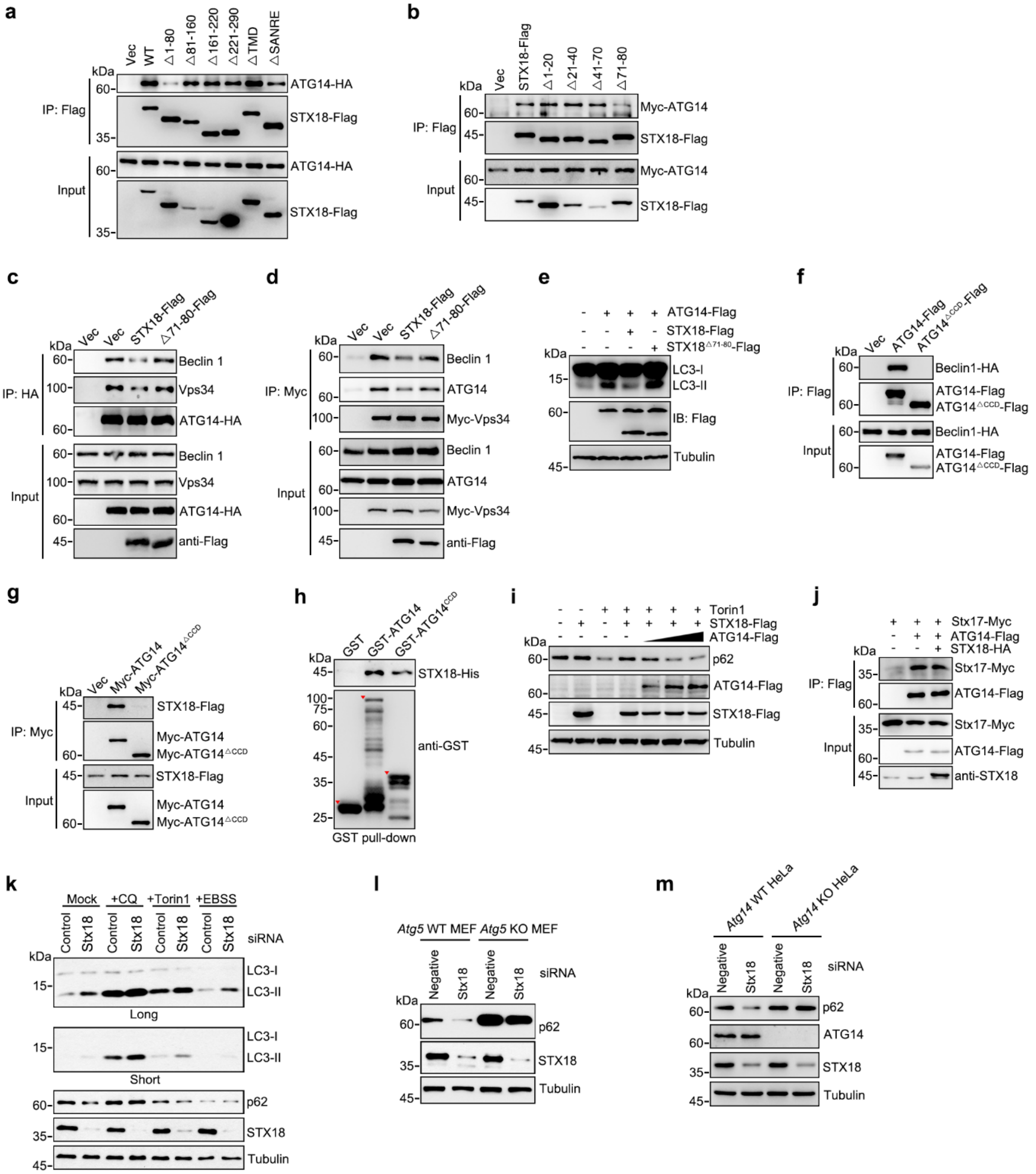
a
Vec
WT
△1-80
△81-160
△161-220
△221-290
△TMD
△SANRE
kDa
IP: Flag
Input
ATG14-HA
STX18-Flag
b
Vec
STX18-Flag
△1-20
△21-40
△41-70
△71-80
Myc-ATG14
c
IP: HA
Beclin 1
Vps34
anti-Flag
d
IP: Myc
ATG14
Myc-Vps34
e
ATG14-Flag
STX18△71-80-Flag
LC3-I
LC3-II
IB: Flag
Tubulin
f
ATG14△CCD-Flag
Beclin1-HA
g
Myc-ATG14
Myc-ATG14△CCD
h
GST
GST-ATG14
GST-ATG14△CCD
STX18-His
anti-GST
GST pull-down
i
Torin1
p62
j
Stx17-Myc
STX18-HA
anti-STX18
k
Mock
+CQ
+Torin1
+EBSS
Control
Stx18
siRNA
Long
Short
STX18
l
Atg5 WT MEF
Atg5 KO MEF
Negative
m
Atg14 WT HeLa
Atg14 KO HeLa

Figure S5. STX18 disrupts the formation of PI3KC3-C1 complex by competitively binding the CCD in ATG14.

(a) HEK293T cells were transfected with ATG14-HA and STX18-Flag or its mutants for 36 h. Protein interactions were detected by immunoprecipitation with anti-Flag beads and immunoblotting analysis. **(b)** HEK293T cells were transfected with Myc-ATG14 and STX18-Flag or its mutants for 36 h. Protein interactions were detected by immunoprecipitation with anti-Flag beads and immunoblotting analysis. **(c)** HEK293T cells were transfected with ATG14-HA and STX18-Flag or its mutant for 36 h. Protein interactions were detected by immunoprecipitation with anti-HA beads and immunoblotting analysis. **(d)** HEK293T cells were transfected with Myc-Vps34 and STX18-Flag or its mutant for 36 h. Protein interactions were detected by immunoprecipitation with anti-Myc beads and immunoblotting analysis. **(e)** HEK293T cells were transfected with ATG14-Flag and STX18-Flag or its mutant for 36 h. Cell lysates were analyzed via western blot. **(f)** HEK293T cells were transfected with Beclin1-HA and ATG14-Flag or $ATG14^{\triangle CCD}$-Flag for 36 h. Protein interactions were detected by immunoprecipitation with anti-Flag beads and immunoblotting analysis. **(g)** HEK293T cells were transfected with STX18-Flag and Myc-ATG14 or Myc-$ATG14^{\triangle CCD}$ for 36 h. Protein interactions were detected by immunoprecipitation with anti-Myc beads and immunoblotting analysis. (**h**) The protein interactions of GST tagged ATG14 or $ATG14^{CCD}$ with His tagged STX18 were detected by GST pull-down experiments. **(i)** HEK293T cells expressing ATG14-Flag and STX18-Flag were treated with Torin1. Cell lysates were analyzed via western blot. **(j)** HEK293T cells were transfected with ATG14-Flag, STX17-Myc, with or without STX18-HA for 36 h. Protein interactions were detected by

immunoprecipitation with anti-Flag beads and immunoblotting analysis. **(k)** U2OS cells were transfected with control or STX18 siRNA for 48 h and then treated with CQ or EBSS starvation for 2 h and treated with Torin1 for 4 h. Cell lysates were analyzed via western blot. **(l)** *Atg5* wild type and knockout MEF were treated with control or STX18 siRNA for 48 h. Cell lysates were analyzed via western blot. **(m)** *Atg14* wild type and knockout HeLa were treated with control or STX18 siRNA for 48 h. Cell lysates were analyzed via western blot. Source data are provided as a Source Data file.

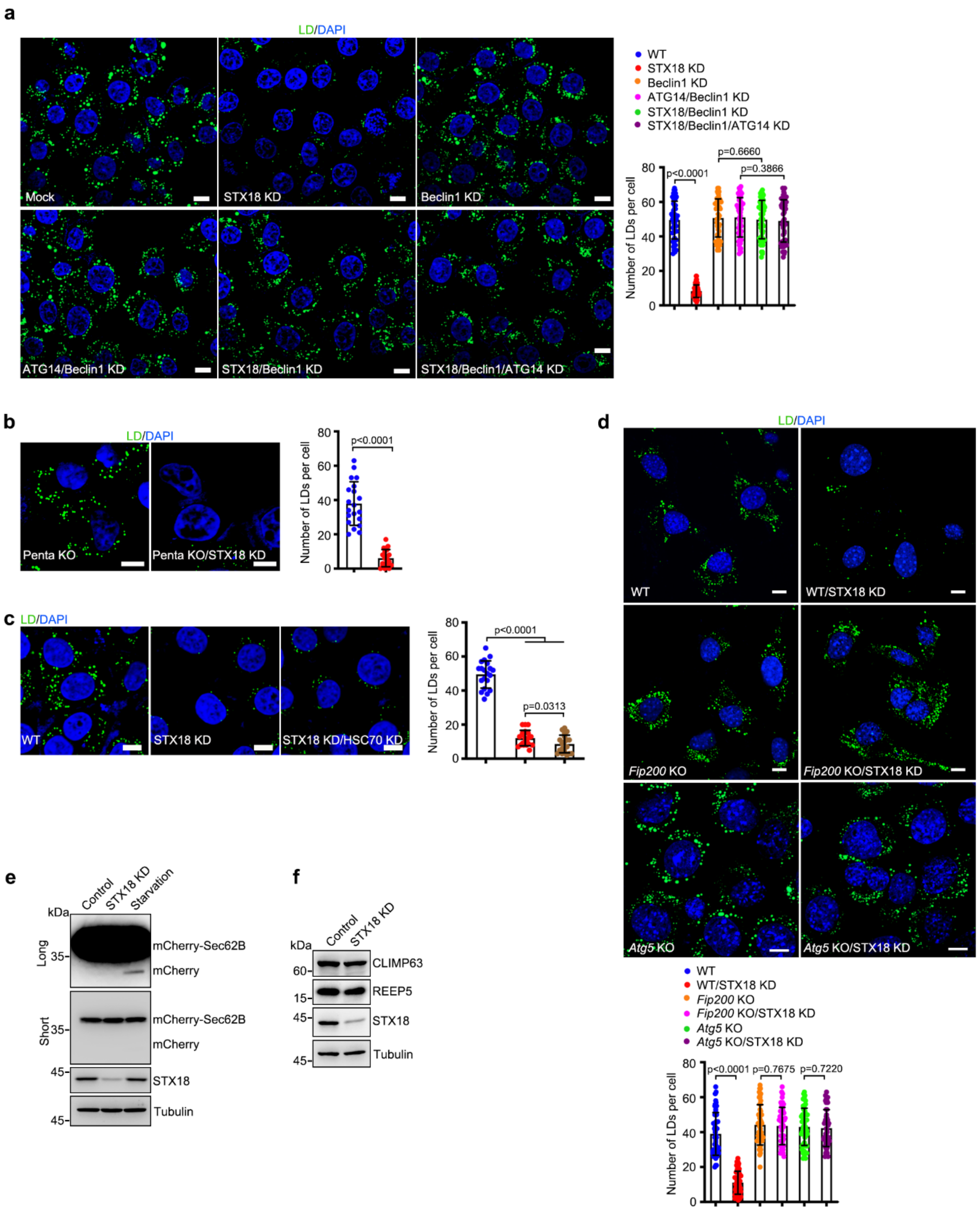
a
LD/DAPI
Mock
STX18 KD
Beclin1 KD
ATG14/Beclin1 KD
STX18/Beclin1 KD
STX18/Beclin1/ATG14 KD
WT
STX18 KD
Beclin1 KD
ATG14/Beclin1 KD
STX18/Beclin1 KD
STX18/Beclin1/ATG14 KD
p<0.0001
p=0.6660
p=0.3866
Number of LDs per cell
b
LD/DAPI
Penta KO
Penta KO/STX18 KD
p<0.0001
Number of LDs per cell
c
LD/DAPI
WT
STX18 KD
STX18 KD/HSC70 KD
p<0.0001
p=0.0313
Number of LDs per cell
d
LD/DAPI
WT
WT/STX18 KD
Fip200 KO
Fip200 KO/STX18 KD
Atg5 KO
Atg5 KO/STX18 KD
WT
WT/STX18 KD
Fip200 KO
Fip200 KO/STX18 KD
Atg5 KO
Atg5 KO/STX18 KD
p<0.0001
p=0.7675
p=0.7220
Number of LDs per cell
e
Control
STX18 KD
Starvation
kDa
Long
Short
mCherry-Sec62B
mCherry
mCherry-Sec62B
mCherry
STX18
Tubulin
f
Control
STX18 KD
kDa
CLIMP63
REEP5
STX18
Tubulin

Figure S6. Knockdown of STX18 induces lipophagy.

(a) HeLa cells were transfected with indicated siRNAs for 48 h, and treated with 200 μM OA for 6 h. LDs were labeled with BODIPY-493/503 (green). The nuclei were stained with DAPI. Cells were imaged by confocal microscopy. Scar bar represents 10 μm. Number of LDs in each cell was counted from 50 cells of three independent experiments. Error bars, mean ± SD. Two-tailed Unpaired Student's t-test. **(b)** *Penta* knockout HeLa cells were transfected with STX18 siRNA for 48 h and treated with 200 μM OA for 6 h. LDs were labeled with BODIPY-493/503 (green). The nuclei were stained with DAPI. Cells were imaged by confocal microscopy. Scar bar represents 10 μm. Number of LDs in each cell was counted from 20 cells of three independent experiments. Error bars, mean ± SD. Two-tailed Unpaired Student's t-test. **(c)** HeLa cells were transfected with STX18 or/and HSC70 siRNA for 48 h and treated with 200 μM OA for 6 h. LDs were labeled with BODIPY-493/503 (green). The nuclei were stained with DAPI. Cells were imaged by confocal microscopy. Scar bar represents 10 μm. Number of LDs in each cell was counted from 20 cells of three independent experiments. Error bars, mean ± SD. Two-tailed Unpaired Student's t-test. **(d)** WT, *Fip200* and *Atg5* KO MEF cells were transfected with si-STX18 for 48 h and treated with 200 μM OA for 6 h. LDs were labeled with BODIPY-493/503 (green). The nuclei were stained with DAPI. Cells were imaged by confocal microscopy. Scar bar represents 10 μm. Number of LDs in each cell was counted from 50 cells of three independent experiments. Error bars, mean ± SD. Two-tailed Unpaired Student's t-test. **(e)** HEK293T cells expressing mCherry-Sec62B were transfected with STX18 siRNA for 48 h or EBSS starvation for 12 h. Cell lysates were analyzed via western

blot. **(f)** HEK293T cells were transfected with STX18 siRNA for 48 h. Cell lysates were analyzed via western blot. Source data are provided as a Source Data file.

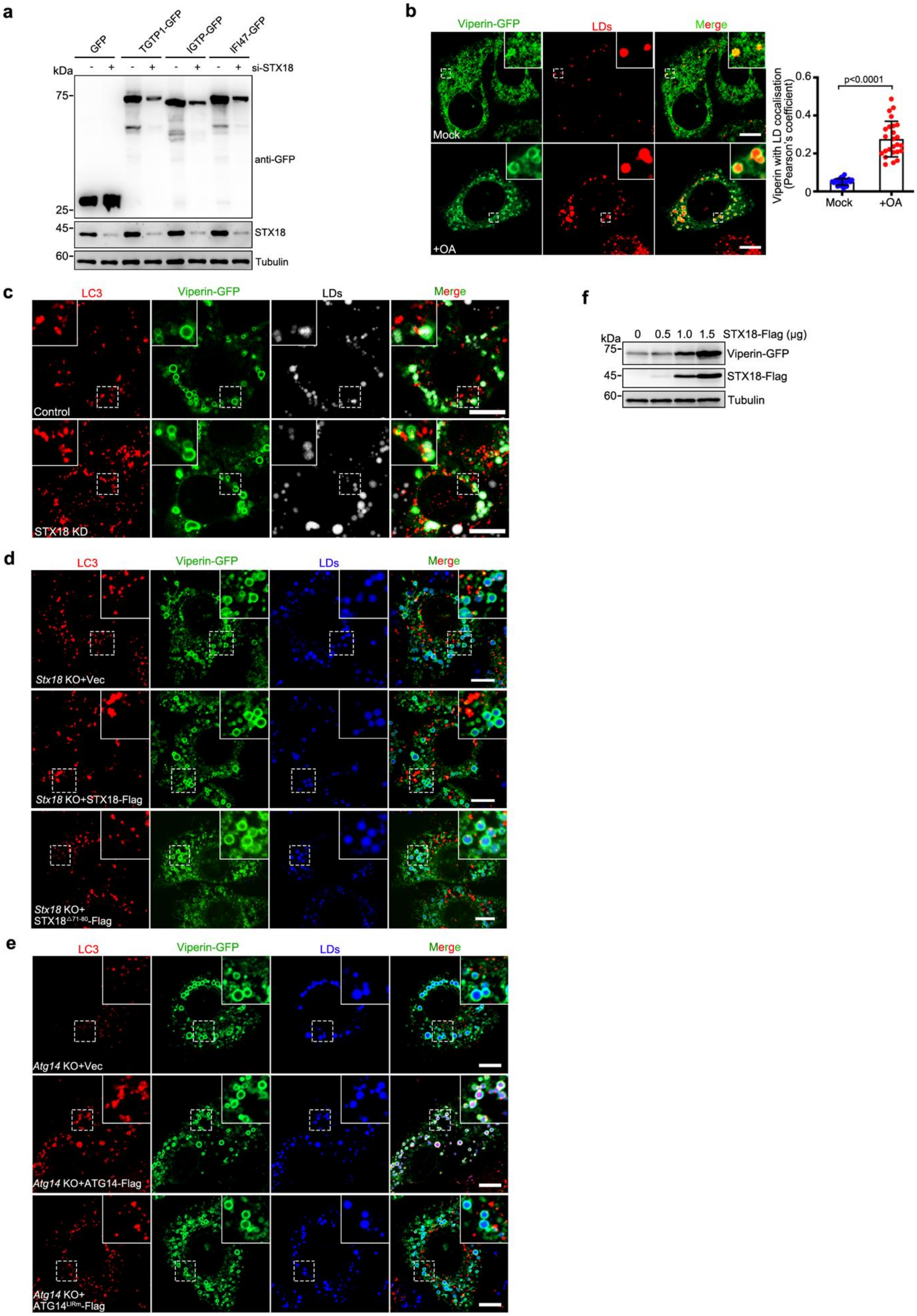

a
GFP
TGTP1-GFP
IGTP-GFP
IFI47-GFP
kDa
- + - + - + - +
si-STX18
75-
25-
anti-GFP
45-
STX18
60-
Tubulin
b
Viperin-GFP
LDs
Merge
Mock
+OA
Viperin with LD cocalisation (Pearson's coefficient)
p<0.0001
0.6
0.4
0.2
0
Mock
+OA
c
LC3
Viperin-GFP
LDs
Merge
Control
STX18 KD
d
LC3
Viperin-GFP
LDs
Merge
Stx18 KO+Vec
Stx18 KO+STX18-Flag
Stx18 KO+
STX18Δ71-80-Flag
e
LC3
Viperin-GFP
LDs
Merge
Atg14 KO+Vec
Atg14 KO+ATG14-Flag
Atg14 KO+
ATG14LIRm-Flag
f
kDa
0 0.5 1.0 1.5 STX18-Flag (µg)
75-
Viperin-GFP
45-
STX18-Flag
60-
Tubulin

Figure S7. STX18 regulates autophagic degradation of Viperin.

(a) HeLa cells expressing GFP-vec, TGTP1-GFP, IGTP-GFP, or IFI47-GFP were transfected with STX18 siRNA for 48 h, and treated with 200 μM OA for 12 h. Cell lysates were analyzed via western blot. **(b)** HeLa cells expressing Viperin-GFP were treated with or without 200 μM OA for 12 h, then fixed and labeled the LDs with LipidTOX Red (red). Cells were imaged by confocal microscopy. Scar bar represents 10 μm. Colocalization of LDs with Viperin-GFP (Pearson's Coefficient), n=25 cells. Error bars, mean ± SD of three independent experiments. Two-tailed Unpaired Student's t-test. **(c)** HeLa cells expressing Viperin-GFP were treated with STX18 siRNA for 48 h. Meanwhile cells were treated with 200 μM OA for 12 h and 100 μM CQ for 6 h, then fixed and immunostained with anti-LC3 antibodies (red). LDs were labeled with LipidTOX Deep Red (white). **(d)** *Stx18* KO HeLa cells expressing Viperin-GFP were transfected with STX18-Flag or STX18$^{\triangle 71\text{-}80aa}$-Flag for 24 h, cells were further treated with 200 μM OA for 12 h and 100 μM CQ for 6 h, then fixed and immunostained with anti-LC3 antibodies (red). LDs were labeled with LipidTOX Deep Red (blue). **(e)** *Atg14* KO HeLa cells expressing Viperin-GFP were transfected with ATG14-Flag or ATG14^{LIRm}-Flag for 24 h, cells were further treated with 200 μM OA for 12 h and 100 μM CQ for 6 h, then fixed and immunostained with anti-LC3 antibodies (red). LDs were labeled with LipidTOX Deep Red (blue). **(f)** HeLa cells were transfected with Viperin-GFP and STX18-Flag for 36 h and treated with 200 μM OA for 12 h. Cell lysates were analyzed via western blot. Source data are provided as a Source Data file.

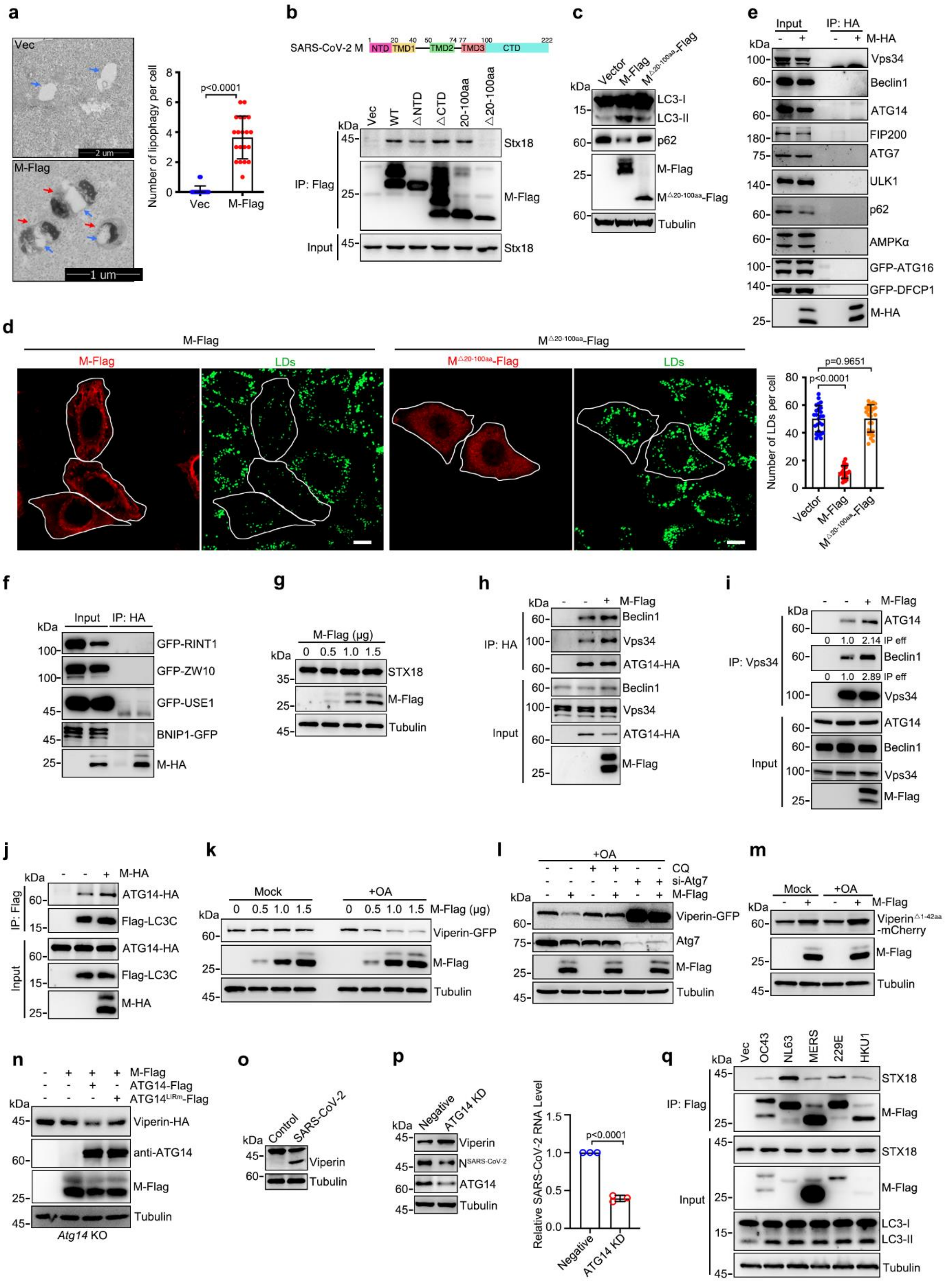
a
Vec
2 um
M-Flag
1 um
Number of lipophagy per cell
p<0.0001
Vec
M-Flag
b
SARS-CoV-2 M
NTD
TMD1
TMD2
TMD3
CTD
1
20
40
50
74
77
100
222
Vec
WT
△NTD
△CTD
20-100aa
△20-100aa
kDa
IP: Flag
Input
Stx18
M-Flag
c
Vector
M-Flag
M△20-100aa-Flag
LC3-I
LC3-II
p62
M△20-100aa-Flag
Tubulin
d
LDs
p=0.9651
p<0.0001
Number of LDs per cell
e
Input
IP: HA
M-HA
Vps34
Beclin1
ATG14
FIP200
ATG7
ULK1
AMPKα
GFP-ATG16
GFP-DFCP1
f
GFP-RINT1
GFP-ZW10
GFP-USE1
BNIP1-GFP
g
M-Flag (μg)
STX18
h
IP: HA
ATG14-HA
i
IP: Vps34
IP eff
j
IP: Flag
Flag-LC3C
k
Mock
+OA
Viperin-GFP
l
CQ
si-Atg7
Atg7
m
Viperin△1-42aa-mCherry
n
ATG14-Flag
ATG14LIRm-Flag
Viperin-HA
anti-ATG14
Atg14 KO
o
Control
SARS-CoV-2
Viperin
p
Negative
ATG14 KD
NSARS-CoV-2
Relative SARS-CoV-2 RNA Level
q
OC43
NL63
MERS
229E
HKU1

Figure S8. SARS-CoV-2 M interacts with STX18 and induces autophagic degradation of Viperin.

(a) Representative transmission electron micrograph of M overexpression cells. Blue arrows indicate LDs. Red arrows indicated autophagosome. The graph shows the quantification of lipophagy by analyzing the number of autophagosomes engulfed LDs per cell in 20 cells. Error bars, mean ± SD of two independent experiments. Two-tailed Unpaired Student's t-test. **(b)** HEK293T cells were transfected with M-Flag or its mutants for 36 h. Cells were subjected to Flag IP and analyzed via western blot. **(c)** HEK293T cells were transfected with M-Flag or its mutant for 36 h. Cell lysates were analyzed via western blot. **(d)** HeLa cells expressing the M-Flag or M$^{\triangle 20\text{-}100aa}$-Flag were treated with 200 μM OA for 6 h, then fixed and immunostained with anti-Flag (red). LDs were labeled with BODIPY-493/503 (green). Cells were imaged by confocal microscopy. White ROIs indicate the cells expressing M protein. Scar bar represents 10 μm. The number of LDs per cell was counted and shown at the right panel, n=25 cells. Error bars, mean ± SD of three independent experiments. Two-tailed Unpaired Student's t-test. **(e)** HEK293T cells were transfected with M-HA, GFP-ATG16, and GFP-DFCP1 for 36 h. Protein interactions were detected by immunoprecipitation with anti-HA beads and immunoblotting analysis. **(f)** HEK293T cells were transfected with M-HA, GFP-RINT1, GFP-ZW10, GFP-USE1 and BNIP1-GFP for 36 h. Protein interactions were detected by immunoprecipitation with anti-HA beads and immunoblotting analysis. **(g)** HEK293T cells were transfected with M-Flag for 36 h. Cell lysates were analyzed via western blot. **(h)** HEK293T cells were transfected with ATG14-HA with or without M-Flag for 36 h. Protein interactions were detected by immunoprecipitation with anti-HA beads and immunoblotting analysis. **(i)** HEK293T cells

were transfected with or without M-Flag for 36 h. Cells were subjected to Vps34 IP and analyzed via western blot. **(j)** HEK293T cells were transfected with Flag-LC3C, ATG14-HA with or without M-HA for 36 h. Protein interactions were detected by immunoprecipitation with anti-Flag beads and immunoblotting analysis. **(k)** HeLa cells were transfected with Viperin-GFP and M-Flag for 36 h and treated with or without 200 μM OA for 12 h. Cell lysates were analyzed via western blot. **(l)** HeLa cells expressing Viperin-GFP with or without M-Flag were transfected with ATG7 siRNA for 48 h or treated with CQ for 6 h. Meanwhile, cells were treated with 200 μM OA for 12 h. Cell lysates were analyzed via western blot. **(m)** HeLa cells expressing Viperin$^{\triangle 1\text{-}42}$-mCherry with or without M-Flag were treated with or without 200 μM OA for 12 h. Cell lysates were analyzed via western blot. **(n)** *Atg14* knockout HeLa cells expressing Viperin-HA with M-Flag were transfected with ATG14-Flag or ATG14^{LIRm}-Flag and treated with 200 μM OA for 12 h. Cell lysates were analyzed via western blot. **(o)** Vero-E6 cells were infected with SARS-CoV-2. Cell lysates were analyzed via western blot. **(p)** Vero-E6 cells were transfected with indicated siRNAs for 24 h and infected with SARS-CoV-2 for 24 h. Meanwhile cells were treated with 200 μM OA for 12 h. Cell lysates were analyzed via western blot. Viral RNA level was determined by RT-qPCR. Error bars, mean ± SD of three independent experiments. Two-tailed Unpaired Student's t-test. **(q)** HEK293T cells were transfected with OC43 M-Flag, NL63 M-Flag, MERS M-Flag, 229E M-Flag, or HKU1 M-Flag for 36 h. Protein interactions were detected by immunoprecipitation with anti-Flag beads and immunoblotting analysis. Source data are provided as a Source Data file.
