# Supplementary material for: ATG14 targets lipid droplets and acts as an autophagic receptor for syntaxin18-regulated lipid droplet turnover
Source: Nat Commun. 2024 Jan 20;15:631. doi: 10.1038/s41467-024-44978-w (PMC10799895; doi:10.1038/s41467-024-44978-w)
Supplement: Supplementary file 3 — Reporting Summary [file 41467_2024_44978_MOESM3_ESM.pdf]

nature portfolio

Corresponding author(s): BINBIN DING

Last updated by author(s): Dec 20, 2023

Reporting Summary

Nature Portfolio wishes to improve the reproducibility of the work that we publish. This form provides structure for consistency and transparency in reporting. For further information on Nature Portfolio policies, see our Editorial Policies and the Editorial Policy Checklist.

Statistics

For all statistical analyses, confirm that the following items are present in the figure legend, table legend, main text, or Methods section.

n/a	Confirmed	
☐	☒	The exact sample size (*n*) for each experimental group/condition, given as a discrete number and unit of measurement
☐	☒	A statement on whether measurements were taken from distinct samples or whether the same sample was measured repeatedly
☐	☒	The statistical test(s) used AND whether they are one- or two-sided<br>*Only common tests should be described solely by name; describe more complex techniques in the Methods section.*
☒	☐	A description of all covariates tested
☒	☐	A description of any assumptions or corrections, such as tests of normality and adjustment for multiple comparisons
☐	☒	A full description of the statistical parameters including central tendency (e.g. means) or other basic estimates (e.g. regression coefficient) AND variation (e.g. standard deviation) or associated estimates of uncertainty (e.g. confidence intervals)
☐	☒	For null hypothesis testing, the test statistic (e.g. *F*, *t*, *r*) with confidence intervals, effect sizes, degrees of freedom and *P* value noted<br>*Give P values as exact values whenever suitable.*
☒	☐	For Bayesian analysis, information on the choice of priors and Markov chain Monte Carlo settings
☒	☐	For hierarchical and complex designs, identification of the appropriate level for tests and full reporting of outcomes
☐	☒	Estimates of effect sizes (e.g. Cohen's *d*, Pearson's *r*), indicating how they were calculated

Our web collection on statistics for biologists contains articles on many of the points above.

Software and code

Policy information about availability of computer code

Data collection	Western blotting images were obtained using Tanon 5200.<br>Confocal images were obtained using LSM780, Zeiss.<br>Transmission electron images were obtained using Hitachi H-7000FA transmission electron microscopy at 80 kV.<br>Immunoelectron images were obtained using Thermo Fisher/FEI Talos L 120C.
Data analysis	Graphing and statistical analyses: GraphPad Prism (v9.5.1) .<br>Immunofluorescence analysis: ImageJ (2.0.0-rc-59/1.51k) and ZEN 3.0 (black edition, 16.0.1.306).<br>Western blotting analysis: Bio-Rad Image Lab Software for PC Version 6.1

For manuscripts utilizing custom algorithms or software that are central to the research but not yet described in published literature, software must be made available to editors and reviewers. We strongly encourage code deposition in a community repository (e.g. GitHub). See the Nature Portfolio guidelines for submitting code & software for further information.

Data

Policy information about availability of data

All manuscripts must include a data availability statement. This statement should provide the following information, where applicable:

- Accession codes, unique identifiers, or web links for publicly available datasets
- A description of any restrictions on data availability
- For clinical datasets or third party data, please ensure that the statement adheres to our policy

All data generated or analyzed during this study are included in this article and its supplemental materials. Source data are provided with this paper.

Research involving human participants, their data, or biological material

Policy information about studies with human participants or human data. See also policy information about sex, gender (identity/presentation), and sexual orientation and race, ethnicity and racism.

Reporting on sex and gender	No human subject research was performed in this study.
Reporting on race, ethnicity, or other socially relevant groupings	N/A
Population characteristics	N/A
Recruitment	N/A
Ethics oversight	N/A

Note that full information on the approval of the study protocol must also be provided in the manuscript.

Field-specific reporting

Please select the one below that is the best fit for your research. If you are not sure, read the appropriate sections before making your selection.

☒ Life sciences ☐ Behavioural & social sciences ☐ Ecological, evolutionary & environmental sciences

For a reference copy of the document with all sections, see nature.com/documents/nr-reporting-summary-flat.pdf

Life sciences study design

All studies must disclose on these points even when the disclosure is negative.

Sample size	Sample size for each experiment is indicated in the legend. No statistical methods were used to predetermine sample sizes. Sample size was chosen based on previous experiments and comparable. The reference of cellular experiments sample size is based on previous experience and comparable experiments (doi: 10.1016/j.celrep.2023.112286 and doi:10.15252/embj.2022112542.).
Data exclusions	No exclusion of data was made only if technical errors were occurred.
Replication	All experimental findings were reproduced in multiple independent experiments. For each figure, the number of independent experiments or biological replicates is indicated in the figure legends or methods section. Western blot and microscopy pictures are from a representative experiment and the number of independent repeats is clearly indicated in the figure legends.
Randomization	For cell culture experiments, cells were split, plated in culture vessels, and then transfected with indicated plasmids or siRNAs. Because control and treatment groups were derived from the same cell line, no randomization could be performed.
Blinding	Investigators were blinded to group allocation during data collection, and not blinded during analyses because the phenotypes (LD number, LD localization) were always significantly changed, it is difficult to blind certain groups when analyzed the fluorescence data; for WB analysis, group informations determines the order of loading samples. No data were excluded during analyses.

Reporting for specific materials, systems and methods

We require information from authors about some types of materials, experimental systems and methods used in many studies. Here, indicate whether each material, system or method listed is relevant to your study. If you are not sure if a list item applies to your research, read the appropriate section before selecting a response.

Materials & experimental systems

n/a	Involved in the study
☐	☒ Antibodies
☐	☒ Eukaryotic cell lines
☒	☐ Palaeontology and archaeology
☒	☐ Animals and other organisms
☒	☐ Clinical data
☒	☐ Dual use research of concern
☒	☐ Plants

Methods

n/a	Involved in the study
☒	☐ ChIP-seq
☒	☐ Flow cytometry
☒	☐ MRI-based neuroimaging

Antibodies

Antibodies used

Mouse anti-Flag (AE005, western blot (WB) 1:5000, immunofluorescence (IF) 1:200), mouse anti-HA (AE008, WB 1:5000, IF 1:200), mouse anti-GFP (AE012, WB 1:5000), mouse anti-GST (AE001, WB 1:5000), mouse anti-His (AE003, WB 1:5000), mouse anti-mCherry (AE002, WB 1:5000), rabbit anti-ATG14 (A7526, WB 1:2000) and rabbit anti-Beclin1 (A7353, WB 1:2000) were obtained from ABclonal. Mouse anti-Tubulin (E7S, WB 1:10000) was obtained from Developmental Studies Hybridoma Bank. Mouse anti-p62 (H00008878-M01, WB 1:10000) was obtained from Abnova. Mouse anti-Myc (2276, WB 1:5000) and rabbit anti-Atg7 (8558, WB 1:5000) were obtained from Cell Signaling Technology. Rabbit anti-LC3 (PM036, WB 1:1000, IF 1:500) was obtained from MBL. Rabbit anti-GFP (ab6556) was obtained from Abcam. Mouse anti-STX18 (sc-293067, WB 1:1000) and mouse anti-GAPDH (sc-365062, WB 1:5000) were obtained from Santa Cruz Biotechnology. Rabbit anti-Vps34 (Z-R015, WB 1:1000) was obtained from Echelon Bioscience. Rabbit anti-ATG14 (19491-1-AP, WB 1:1000), rabbit anti-Viperin (28089-1-AP, WB 1:1000), rabbit anti-CLIMP63 (16686-1-AP, WB 1:1000) and rabbit anti-REEP5 (14643-1-AP, WB 1:1000) were obtained from Proteintech. Mouse anti-SARS-CoV-2 Nucleocapsid (N) (40143-MM05, WB 1:1000) was obtained from Sino Biological Inc. Mouse anti-VSV-M (EB0011, WB 1:1000) was obtained from kerafast. HRP-conjugated goat anti-mouse IgG (H+L) (AS003, WB 1:10000), HRP-conjugated goat anti-rabbit IgG (H+L) (AS014, WB 1:10000) were obtained from Abclonal. Peroxidase-AffiniPure goat anti-mouse IgG light-chain-specific (115-035-174, WB 1:10000) and peroxidase IgG fraction monoclonal mouse anti-Rabbit IgG, light-chain-specific (211-032-171, WB 1:10000) were obtained from Jackson Immunoresearch. Alexa Fluor 488-conjugated goat anti-mouse IgG (H+L) (A32723, IF 1:500), goat anti-rabbit IgG (H+L, IF 1:500) (A32731), Alexa Fluor 568-conjugated goat anti-mouse IgG (H+L) (A-11031, IF 1:500), goat anti-rabbit IgG (H+L) (A-11036, IF 1:500), Alexa Fluor 647-conjugated goat anti-mouse IgG (H+L) (A-21236, IF 1:500) and goat anti-rabbit IgG (H+L) (A-21244, IF 1:500) were purchased from Thermo Fisher Scientific.

Validation

All primary antibodies were obtained from indicated commercial vendors with ensured quality and were validated by the companies from which they were purchased or by previous studies.
Citations are listed as below:
anti-Flag (ABclonal, AE005): https://abclonal.com.cn/catalog/AE005
anti-HA (ABclonal, AE008): https://abclonal.com.cn/catalog/AE008
anti-GFP (ABclonal, AE012): https://abclonal.com.cn/catalog/AE012
anti-GST (ABclonal, AE001): https://abclonal.com.cn/catalog/AE001
anti-His (ABclonal, AE003): https://abclonal.com.cn/catalog/AE003
anti-mCherry (ABclonal, AE002): https://abclonal.com.cn/catalog/AE002
anti-ATG14 (ABclonal, A7526): https://abclonal.com.cn/catalog/A7526
anti-Beclin1 (ABclonal, A7353): https://abclonal.com.cn/catalog/A7353
anti-Tubulin (Developmental Studies Hybridoma Bank, E7S) was validated in human through WB: Diao, J.J. et al. ATG14 promotes membrane tethering and fusion of autophagosomes to endolysosomes. Nature 520, 563-6 (2015).
anti-p62 (Abnova, H00008878-M01): https://www.abnova.com/products/products_detail.asp?catalog_id=H00008878-M01
anti-Myc (CST #2276, 9B11): https://www.cellsignal.cn/products/primary-antibodies/myc-tag-9b11-mouse-mab/2276
anti-Atg7 (CST #8558, D12B11): https://www.cellsignal.cn/products/primary-antibodies/atg7-d12b11-rabbit-mab/8558
anti-LC3 (MBL, PM036): https://ruo.mbl.co.jp/bio/dtl/dtlfiles/PM036-v12.pdf
anti-GFP (Abcam, ab6556): https://www.abcam.cn/products/primary-antibodies/gfp-antibody-ab6556.html
anti-STX18 (Santa Cruz Biotechnology, sc-293067): https://www.scbt.com/p/syntaxin-18-antibody-10
anti-GAPDH (Santa Cruz Biotechnology, sc-365062):
https://www.scbt.com/p/gapdh-antibody-g-9
anti-Vps34 (Echelon Bioscience, Z-R015): https://www.echelon-inc.com/?s=Z-R015
anti-ATG14 (Proteintech, 19491-1-AP): https://www.ptgcn.com/products/Barkor-Specific-Antibody-19491-1-AP.htm
anti-Viperin (Proteintech, 28089-1-AP):
https://www.ptgcn.com/products/RSAD2-Antibody-28089-1-AP.htm
anti-CLIMP63 (Proteintech, 16686-1-AP): https://www.ptgcn.com/products/CKAP4-Antibody-16686-1-AP.htm
anti-REEP5 (Proteintech, 14643-1-AP): https://www.ptgcn.com/products/REEP5-Antibody-14643-1-AP.htm
anti-SARS-CoV-2 Nucleocapsid (N) (Sino Biological Inc, 40143-MM05): https://www.sinobiological.com/antibodies/cov-nucleocapsid-40143-mm05
anti-VSV-M (kerafast, EB0011): http://kerafast.com.cn/index.php?id=1929&project=product
HRP Goat Anti-Mouse IgG (H+L) (ABclonal, AS003): https://abclonal.com.cn/catalog/AS003
HRP Goat Anti-Rabbit IgG (H+L) (ABclonal, AS014): https://abclonal.com.cn/catalog/AS014
Peroxidase-AffiniPure goat anti-mouse IgG light-chain-specific (Jackson Immunoresearch, 115-035-174): https://www.jacksonimmuno.com/catalog/products/115-035-174
Peroxidase IgG fraction monoclonal mouse anti-Rabbit IgG, light-chain-specific (Jackson Immunoresearch, 211-032-171): https://www.jacksonimmuno.com/catalog/products/211-032-171
Alexa Fluor 488-conjugated goat anti-mouse IgG (H+L) (Thermo Fisher Scientific, A32723): https://www.thermofisher.cn/cn/zh/antibody/product/Goat-anti-Mouse-IgG-H-L-Highly-Cross-Adsorbed-Secondary-Antibody-Polyclonal/A32723
Alexa Fluor 488-conjugated goat anti-rabbit IgG (H+L) (Thermo Fisher Scientific, A32731): https://www.thermofisher.cn/cn/zh/

antibody/product/Goat-anti-Rabbit-IgG-H-L-Highly-Cross-Adsorbed-Secondary-Antibody-Polyclonal/A32731
Alexa Fluor 568-conjugated goat anti-mouse IgG (H+L) (Thermo Fisher Scientific, A-11031): https://www.thermofisher.cn/cn/zh/antibody/product/Goat-anti-Mouse-IgG-H-L-Highly-Cross-Adsorbed-Secondary-Antibody-Polyclonal/A-11031
Alexa Fluor 568-conjugated goat anti-rabbit IgG (H+L) (Thermo Fisher Scientific, A-11036): https://www.thermofisher.cn/cn/zh/antibody/product/Goat-anti-Rabbit-IgG-H-L-Highly-Cross-Adsorbed-Secondary-Antibody-Polyclonal/A-11036
Alexa Fluor 647-conjugated goat anti-mouse IgG (H+L) (Thermo Fisher Scientific, A-21236): https://www.thermofisher.cn/cn/zh/antibody/product/Goat-anti-Mouse-IgG-H-L-Highly-Cross-Adsorbed-Secondary-Antibody-Polyclonal/A-21236
Alexa Fluor 647-conjugated goat anti-rabbit IgG (H+L) (Thermo Fisher Scientific, A-21244): https://www.thermofisher.cn/cn/zh/antibody/product/Goat-anti-Rabbit-IgG-H-L-Cross-Adsorbed-Secondary-Antibody-Polyclonal/A-21244

Eukaryotic cell lines

Policy information about cell lines and Sex and Gender in Research

Cell line source(s)	HEK293T cells (CRL-1573), HeLa cells (CCL-2) and U2OS cells (HTB-96) were obtained from ATCC.<br>BHK21 cells were obtained from Mingzhou Chen (Wuhan University).<br>Vero-E6 cells were obtained from Kun Cai (Hubei Provincial Center for Disease Control and Prevention).<br>HepG2, WT MEF, Fip200 KO MEF, and Atg5 KO MEF cells were obtained from Qing Zhong (Shanghai Jiao Tong University).<br>HEK293F cells were obtained from Hongjun Yu (Huazhong University of Science and Technology).<br>Penta KO HeLa cells were obtained from Richard J. Youle (NIH).<br>Stx18 WT and KO HEK293T, Stx18 WT and KO HeLa, Atg14 WT and KO HeLa, Atg14 WT and KO Vero-E6 were constructed in our lab.
Authentication	Cells were not authenticated.
Mycoplasma contamination	Cell lines have been tested negative for mycoplasma contamination.
Commonly misidentified lines<br>(See ICLAC register)	There is no any commonly misidentified cell lines in this study.
